# Using an Extended Technology Acceptance Model to Understand the Factors Influencing Telehealth Utilization After Flattening the COVID-19 Curve in South Korea: Cross-sectional Survey Study

**DOI:** 10.2196/25435

**Published:** 2021-01-08

**Authors:** Min Ho An, Seng Chan You, Rae Woong Park, Seongwon Lee

**Affiliations:** 1 So-Ahn Public Health Center Jeon-ra-nam-do Republic of Korea; 2 Department of Biomedical Informatics Ajou University School of Medicine Suwon Republic of Korea; 3 Department of Biomedical Sciences Ajou University Graduate School of Medicine Suwon Republic of Korea

**Keywords:** telemedicine, telehealth, COVID-19, pandemic, model, South Korea, acceptance, anxiety, cross-sectional

## Abstract

**Background:**

Although telehealth is considered a key component in combating the worldwide crisis caused by COVID-19, the factors that influence its acceptance by the general population after the flattening of the COVID-19 curve remain unclear.

**Objective:**

We aimed to identify factors affecting telehealth acceptance, including anxiety related to COVID-19, after the initial rapid spread of the disease in South Korea.

**Methods:**

We proposed an extended technology acceptance model (TAM) and performed a cross-sectional survey of individuals aged ≥30 years. In total, 471 usable responses were collected. Confirmatory factor analysis was used to examine the validity of measurements, and the partial least squares (PLS) method was used to investigate factors influencing telehealth acceptance and the impacts of COVID-19.

**Results:**

PLS analysis showed that increased accessibility, enhanced care, and ease of telehealth use had positive effects on its perceived usefulness (*P*=.002, *P*<.001, and *P*<.001, respectively). Furthermore, perceived usefulness, ease, and privacy/discomfort significantly impacted the acceptance of telehealth (*P*<.001, *P*<.001, and *P*<.001, respectively). However, anxiety toward COVID-19 was not associated with telehealth acceptance (*P*=.112), and this insignificant relationship was consistent in the cluster (n=216, 46%) of respondents with chronic diseases (*P*=.185).

**Conclusions:**

Increased accessibility, enhanced care, usefulness, ease of use, and privacy/discomfort are decisive variables affecting telehealth acceptance in the Korean general population, whereas anxiety about COVID-19 is not. This study may lead to a tailored promotion of telehealth after the pandemic subsides.

## Introduction

### Background

The COVID-19 pandemic, caused by SARS-CoV-2 infection, has changed the world in various ways. Due to the highly contagious nature of this novel virus and shortages in personal protective equipment, health care centers have become high-risk transmission areas, and health care workers are at high risk for contracting COVID-19 [1]. In China where the first COVID-19 outbreak was documented, a significant proportion of cases were due to hospital-related transmission [2]. Accordingly, many studies reported that visits to health care centers had been dramatically decreased during the initial phase of the pandemic [3,4]. As a result, telehealth has gained unprecedented attention in the world as a protective measure against COVID-19 [5].

As a country situated close to China, South Korea was soon affected with its own outbreak. The first patient with a confirmed COVID-19 diagnosis entered the country on January 19, 2020 [6]. The outbreak was augmented by a religious gathering in Daegu, a city in southeastern South Korea [7]. The number of confirmed cases dramatically increased and reached a count of 909 cases daily on February 29, which was the highest number of cases reported by far in South Korea [8]. Meanwhile, rapid nationwide screening for COVID-19 was conducted alongside social distancing, mask use, and temporary implementation of telehealth. From February 24 to April 12, a total of 103,998 telehealth appointments were conducted in South Korea (2167 appointments per day on average) [9]. As of early June, the average number of daily incident cases was 55 in South Korea during an entire week, which represents a remarkable decrease from the previous average number of daily cases of 445 between February 25 and March 10 (15 days).

Kidholm et al [10] defined telehealth as “the delivery of health care services through the use of information and communication technologies in a situation where the actors are at different locations.” Rho et al [11] stated that telehealth is “the interchange of health information using telecommunications technology by geographically disconnected providers and patients with the intention to evaluate, diagnose, treat, or educate the patient.” In this study, we defined telehealth as health care services for diagnosis, treatment, or counseling delivered via telecommunication technologies by medical professionals at remote locations.

Although there is little doubt about the considerable benefit of telehealth in terms of managing the crisis caused by COVID-19 [12], the long-term prospect of telehealth remains largely unclear. However, there are conflicting opinions on the continuation of telehealth use after COVID-19. Some argue that telehealth may be abandoned after the COVID-19 curve is flattened [13,14]. Meanwhile, in Israel, the increase in the use of phone visits in pediatric clinics was sustained after lockdown restrictions were lifted [14]. Therefore, we attempted to predict trends in health care service use after COVID-19 by investigating the impact of the disease on the acceptance of telehealth.

In this study, we aimed to identify factors affecting the acceptance of telehealth by performing a survey of the Korean general population. Furthermore, we investigated whether anxiety related to COVID-19 had any significant impact on telehealth acceptance.

### Research Model

According to the technology acceptance model (TAM), usefulness and easiness are the two major factors involved in user adoption of a technology [15]. TAM has been widely used to evaluate user acceptance of general technologies but is limited by little explanatory power for specific system purposes [16]. Therefore, to evaluate the acceptance of telehealth, we extended TAM with predicted benefits and concerns for telehealth.

Hirani et al [17] studied user beliefs on telehealth acceptance and presented the following constructs regarding its precedents and consequences: (1) enhanced care, (2) increased accessibility, (3) privacy and discomfort, (4) care personnel concerns, (5) kit as substitution, and (6) satisfaction. Enhanced care and increased accessibility are benefits that telehealth may provide to patients, whereas privacy and discomfort, as well as care personnel concerns, are obstacles that may hinder telehealth acceptance. Kit as substitution refers to one’s beliefs about how telehealth may be an alternative to regular care, and satisfaction is the gratification experienced as a result of the telehealth system and service. Among them, we selected three precedent variables, namely increased accessibility, enhanced care, and privacy and discomfort, since this study aimed to explore the factors influencing the acceptance of the telehealth system itself. The care personnel concerns construct was excluded as a variable because it indicates concerns about the capabilities of the health care provider and does not pertain to the telehealth system.

**Figure 1:**
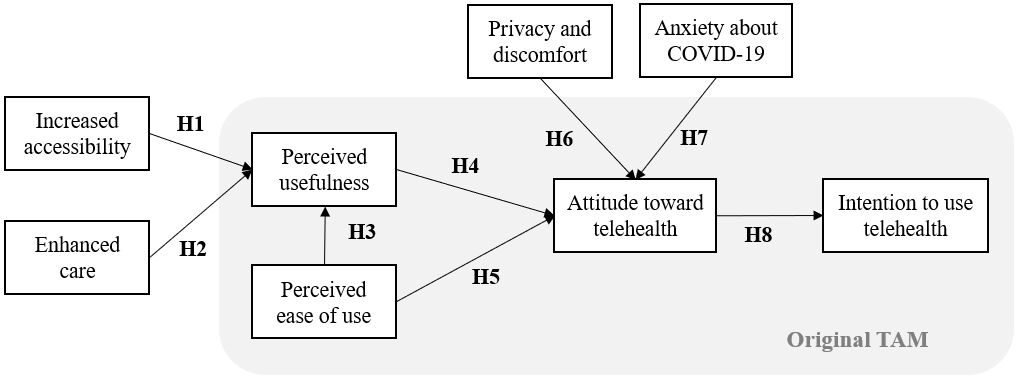
Research model. TAM: technology acceptance model.

In addition, to study the impact of COVID-19 on telehealth acceptance, we included the construct of anxiety related to COVID-19 in the research model (Figure 1).

### Development of Hypotheses

#### Telehealth Usefulness

Increased accessibility is a key element for the success of health care services. Accessibility is the belief pertaining to how a health care system has facilitated the receipt of care from health care providers [17]. Access to health care is the interplay between the characteristics of persons, and social and physical environments, and the characteristics of health systems, institutions, and providers, and it plays a central role in the performance of a health care system [16]. Facilitating access to health care increases the opportunity to obtain appropriate care services in situations where it is needed, and enhances the utilization of such services in terms of service availability and relevance, as well as physical and financial accessibility [18].

With telehealth, patients do not need to travel to the hospital and wait to see their physician [19]. Moreover, telehealth makes it possible for disabled patients and patients with other barriers to care, such as those who are housebound or live in rural areas, to access services [20]. The accessibility of telehealth will increase the usefulness of telehealth and thus we hypothesized:

H1. Increased accessibility has a positive impact on the perceived usefulness of telehealth.

Enhanced care is defined as one’s beliefs on how telehealth can improve the care that patients receive from their health care professional [17]. Telehealth makes it easier for patients to consult health care professionals and increases the possibility of seamless health care and early detection of diseases [17]. It may improve the efficiency of health care in terms of convenience in follow-up while maintaining clinical effectiveness with less cost for both patients and clinicians compared with traditional visits [21]. Telehealth was also found to be effective in certain fields, including psychological interventions [22] and home monitoring of respiratory conditions [23], and for chronic diseases including diabetes, heart disease, and chronic obstructive pulmonary diseases [24,25]. This enhanced health care system will increase people’s perceptions of the usefulness of telehealth; thus, we hypothesized the following:

H2. Enhanced care has a positive impact on the perceived usefulness of telehealth.

TAM asserts that user perception regarding the usefulness of a technology is influenced by its ease of use. Perceived ease of use refers to the extent to which a person believes that using the system will be free of effort [15]. The easier the system is to use, the more useful it can be [26]. For telehealth, the ease of use will also increase the perceived usefulness of it, and thus we hypothesized:

H3. Perceived ease of use has a positive impact on the perceived usefulness of telehealth.

#### Attitude Toward Telehealth

TAM stipulates that perceived usefulness and perceived ease of use are factors associated with people’s attitude toward a system [15]. The attitude toward telehealth is defined by positive or negative feelings related to using a telehealth service [27]. According to the theory of reasoned action, people’s beliefs such as perceived usefulness and perceived ease of use shapes an attitude, which, in turn, influences a behavior [28]. Many studies have demonstrated that when people perceive a technology as useful, the likelihood of accepting it increases [29,30]. Evidence also shows that when a technology is easy to use, the attitude toward it improves [31]. We anticipated that the perceived usefulness and the perceived ease of use of telehealth would improve people’s attitude toward it. We developed the following two hypotheses:

H4. Perceived usefulness has a positive impact on attitude toward telehealth.

H5. Perceived ease of use has a positive impact on attitude toward telehealth.

Privacy and discomfort are major concerns that hinder telehealth adoption [16]. This construct can be defined as concerns about the impact of telehealth on the safety of personal and health information [17]. Generally, telehealth involves the digital collection, use, disclosure, and communication of health information over a network between health care providers and patients [32]. Health information is highly confidential, so people may experience concerns about privacy intrusions and loss of control over information [19,33,34]. To realize the potential of telehealth, trust between health care providers and patients without privacy concerns is required. The greater the concern regarding privacy and discomfort related to the use of telehealth, the worse the attitude toward telehealth, and thus we hypothesized:

H6. Privacy and discomfort have a negative impact on attitude toward telehealth.

The COVID-19 pandemic may provide an increased incentive for telehealth use [5]. People have been subjected to a number of public policies such as regional lockdowns, quarantine at home, physical distancing, and restricted travel [35,36]. They are concerned about hospital visits because of the probability of contracting COVID-19 in this setting, which can lead to serious complications, especially for patients with chronic diseases.

In a study on the adoption of Google Meet for education, students’ perceived fear of COVID-19 significantly affected the intention to attend the class via Google Meet [37]. This finding is relevant to our paper in that it supports the idea that psychological factors can affect the behavior of users.

A recent study investigating panic during the COVID-19 pandemic in the Philippines using the Health Anxiety Inventory reported that levels of avoidance behavior and symptoms of hypochondriasis differed between residents inside and outside Metro Manila [38]; this implies that anxieties about contracting COVID-19 may alter the behavior of the public.

Additionally, a study from China reported that approximately one-third of the survey participants reported having moderate to severe anxiety, with 84.7% of respondents spending most of their time at home and 75.2% worrying about their family members being exposed to COVID-19 [39]. Therefore, people who are anxious about COVID-19 will be more positive about accepting non–face-to-face health care services. Thus, we hypothesized:

H7. Anxiety related to COVID-19 has a positive impact on attitude toward telehealth.

#### Intention to Use Telehealth

The intention to use telehealth is defined by the extent to which a population intends to use telehealth [11]. According to TAM, the intention to use a technology is influenced by one’s attitude toward it [16]; this intention predicts the actual usage behavior [31]. A positive attitude, including high favorability, and satisfaction of a technology results increase one’s intention to use it; hence, a positive attitude toward telehealth increases the intention to use it. Therefore, we hypothesized:

H8. Attitude toward telehealth has a positive impact on the intention to use telehealth.

## Methods

### Measurement Instruments

To ensure the validity of the measures, all measurement items for each variable in the model were developed based on previous studies. We modified them to measure the perceptions and attitudes toward telehealth. A questionnaire originally developed in English was translated into Korean and was repeatedly examined to ensure that the items and expressions in both versions were consistent.

The questionnaire consisted of three parts. The first part pertained to perceptions and beliefs regarding telehealth, including the TAM variables. The second part included questions on anxiety level in relation to COVID-19, and the last part included questions on respondents’ sociodemographic information (eg, gender, age group, education level, monthly income), hospital usage patterns (eg, frequency of hospital visits), and their health status (eg, comorbidities).

Variables related to beliefs about telehealth, increased accessibility, enhanced care, and privacy and discomfort were measured using the Service User Technology Acceptability Questionnaire by Hirani et al [17]. Four items were used to measure each respondent’s increased accessibility to telehealth, and 5 items were used for enhanced care. For privacy and discomfort, there were initially 4 items, but one was removed during the reliability test, resulting in 3 items.

The TAM variables of perceived usefulness of, perceived ease of use of, and intention to use telehealth were developed from measurement items published by Venkatesh and Davis [26]. Perceived ease of use was measured with 4 measurement items, and 2 items were used to measure intention to use telehealth. For perceived usefulness, 4 items were used, but one was removed during the reliability test, and the remaining 3 items were used for analysis. Attitude toward telehealth was measured by 4 questions, which were developed from Davis [15].

Anxiety about COVID-19 was measured using items published by Roy et al [40]. They developed 18 items to measure people’s feelings of anxiety toward COVID-19 based on a 5-point Likert scale (1=never, 2=rarely, 3=sometimes, 4=often, 5=always). We sorted these items in the order of the highest number of answers of “often” or “always,” and selected 6 items for which over 80% of respondents had answered as “often” or “always.” During the reliability test, 3 items were removed, and a total of 3 items were included for analysis. The detailed items of each construct are listed in Table 1; each item was measured by a 5-point Likert scale.

**Table 1:** Measurement items of constructs^a^.

Construct and item		Measurements	Reference
**Increased accessibility (AC)**			Hirani et al [17]
	ac1	Telehealth increases my access to health care.	
	ac2	Telehealth helps me to improve my health.	
	ac3	Telehealth saves me time in that I do not have to visit my GP^b^ clinic.	
	ac4	Telehealth has made it easier to get in touch with health care professionals.	
**Enhanced care (EC)**			Hirani et al [17]
	ec1	Telehealth makes me actively involved in my health.	
	ec2	Telehealth allows the people looking after me to better monitor me and my condition.	
	ec3	Telehealth can be recommended to people with a similar condition to mine.	
	ec4	Telehealth can certainly be a good addition to regular health care.	
	ec5	Telehealth allows me to be less concerned about my health care.	
**Perceived ease of use (PE)**			Venkatesh and Davis [26]
	pe1	My interaction with telehealth is clear and understandable.	
	pe2	Interacting with telehealth does not require a lot of mental effort.	
	pe3	I find telehealth to be easy to use.	
	pe4	I find it easy to get the telehealth system to do what I want it to do.	
**Perceived usefulness (PU)**			Venkatesh and Davis [26]
	pu2	Using telehealth in my job increases my productivity of health care.	
	pu3	Using telehealth enhances the effectiveness of my health care.	
	pu4	I find telehealth to be useful to my health care.	
**Privacy and discomfort (PD)**			Hirani et al 17]
	pd1	Telehealth makes me feel uncomfortable physically or emotionally.	
	pd2	The telehealth service I received invades my privacy.	
	pd3	The telehealth service I received interferes with my everyday routine.	
**COVID-19–related anxiety (CA)**			Roy et al [40]
	ca2	Since last week, how often have you avoided partying?	
	ca3	Since last week, how often have you avoided social contact?	
	ca4	Since last week, how often have you avoided large meetings and gatherings?	
**Attitude (AT)**			Davis [15]
	at1	Using telehealth is a good idea.	
	at2	Using telehealth is a wise idea.	
	at3	I like using telehealth.	
	at4	Using telehealth makes me feel good.	
**Intention to use (UI)**			Venkatesh and Davis [26]
	ui1	Assuming I have access to telehealth, I intend to use it.	
	ui2	Given that I have access to telehealth, I predict that I would use it.	

^a^Items for each variable, except anxiety about COVID-19, were measured on a 5-point Likert scale (1=totally disagree to 5=totally agree). Items for anxiety about COVID-19 were also measured on a 5-point Likert scale but using the following designations: 1=never, 2=rarely, 3=sometimes, 4=often, and 5=always.

^b^GP: general practitioner.

### Data Collection

Data were collected through a cross-sectional survey. We used a mobile survey company, OpenSurvey, to recruit participants and collect questionnaire data. Using OpenSurvey’s panel and smartphone app, data could be collected nationwide. We included only individuals aged ≥30 years. To reduce confounding effects, stratified sampling was used for 4 age groups: 30-39, 40-49, 50-59, and ≥60 years. The survey was conducted on July 3, 2020, when the average number of daily confirmed cases of COVID-19 was approximately 50 per week (June 29 to July 5) after the initial rapid spread of COVID-19 in South Korea. The questionnaire was distributed to a panel that met the study criteria, and 500 responses were collected. In order to encourage participation, USD 0.84 (KRW 1000) was paid to each questionnaire respondent.

This study was approved by the Ethics Committee of Ajou University (AJIRB-SBR-SUR-20-227), South Korea.

### Data Analysis

The partial least squares (PLS) method, based on structural equation modeling, was used to validate the research model. First, we evaluated the validity and internal consistency of research constructs with measurement analysis: factor loading, the average variance extracted (AVE), and Cronbach alpha. Second, we performed PLS analysis to validate our hypotheses. SmartPLS 3.0 (SmartPLS GmbH) was used as a statistical analytic software.

## Results

### Demographic Characteristics

The total number of collected questionnaires was 500. Of these, 29 were excluded since the respondents provided the same answer to all questionnaire items. Data from 471 respondents were included for analysis. Table 2 shows the respondents’ demographic information. A total of 232 (49.26%) respondents were male, and respondents were almost equally distributed across the age groups. Many respondents had received an education equivalent to a bachelor’s degree (n=300, 63.69%). The most commonly reported income in our study population was $2000-$3000 (n=112, 23.78%) and $3000-$4000 (n=96, 20.38%). Only 16 (3.40%) participants had used telehealth during the past year. Some respondents had major chronic diseases, such as hypertension (n=70, 14.86%), diabetes (n=31, 6.58%), and heart disease (n=28, 5.94%), and 193 (40.98%) participants reported that they had visited the hospital 3-6 times a year.

**Table 2:** Demographics of respondents (N=471).

Characteristic		Participant, n (%)
**Gender**		
	Male	232 (49.26)
	Female	239 (50.74)
**Age group (years)**		
	30-39	119 (25.27)
	40-49	115 (24.42)
	50-59	116 (24.63)
	60-69	121 (25.69)
**Education**		
	High school education or lower	10 (2.12)
	High school graduate	112 (23.78)
	Bachelor’s degree	300 (63.69)
	Master’s degree or other	49 (10.4)
**Income per month**		
	<$1000	53 (11.25)
	$1000-$2000	73 (15.50)
	$2000-$3000	112 (23.78)
	$3000-$4000	96 (20.38)
	$4000-$5000	64 (13.59)
	>$5000	73 (15.50)
**Telehealth experience**		
	No	455 (96.60)
	Yes	16 (3.40)
**Number of hospital visits per year**		
	<3	179 (38.00)
	3-6	193 (40.98)
	7-12	78 (16.56)
	≥13	21 (4.46)
**Chronic disease**		
	Hypertension	70 (14.86)
	Diabetes	31 (6.58)
	Cancer	10 (2.12)
	Stroke	4 (0.85)
	Heart disease	28 (5.94)
	Depression	20 (4.25)
	Asthma	17 (3.61)
	Other	36 (7.64)

### Measurement Model

We used reflective measurement modeling for all 8 latent variables, in which indicators are influenced by the variables not composing them [41]. First, the reliability and convergent validity of the measurement model was evaluated by confirmatory factor analysis. As a result of factor loading, the items with a loading value not exceeding 0.7 were excluded from the analysis [42]. Those items were 1 for privacy and discomfort and 3 for anxiety about COVID-19. The internal consistency of the constructs was examined by a Cronbach alpha coefficient greater than .7, which is an accepted cut-off [43,44]. The AVEs of all the constructs were well above 0.50, and the convergent validity of the measurement items was validated [45]. Table 3 shows the results of the factor loadings, composite reliability, AVE, and Cronbach alpha.

**Table 3:** Factor loadings and reliability.

Construct and item			Mean (SD)		Loadings		Composite reliability		Average variance extracted		Cronbach alpha
**Increased accessibility (AC)**							0.895		0.680		.842
	ac1	3.979 (0.860)		0.877							
	ac2	3.798 (0.863)		0.860							
	ac3	4.244 (0.765)		0.779							
	ac4	3.989 (0.886)		0.779							
**Enhanced care (EC)**							0.919		0.695		.890
	ec1	3.786 (0.891)		0.823							
	ec2	3.272 (0.995)		0.779							
	ec3	3.626 (0.885)		0.864							
	ec4	3.885 (0.855)		0.875							
	ec5	3.673 (0.911)		0.825							
**Perceived ease of use (PE)**							0.891		0.673		.838
	pe1	3.325 (0.917)		0.815							
	pe2	3.316 (0.972)		0.742							
	pe3	3.505 (0.913)		0.875							
	pe4	3.667 (0.907)		0.842							
**Perceived usefulness (PU)**							0.913		0.779		.858
	pu2	3.735 (0.858)		0.878							
	pu3	3.463 (0.919)		0.871							
	pu4	3.756 (0.914)		0.897							
**Privacy and discomfort (PD)**							0.908		0.767		.848
	pd1	2.204 (0.890)		0.852							
	pd2	2.293 (0.936)		0.879							
	pd3	2.055 (0.881)		0.897							
**Anxiety about COVID-19 (CA)**							0.867		0.684		.769
	ca2	2.291 (1.363)		0.814							
	ca3	3.123 (1.235)		0.822							
	ca4	2.713 (1.635)		0.845							
**Attitude (AT)**							0.933		0.778		.904
	at1	3.662 (0.942)		0.857							
	at2	3.675 (0.919)		0.928							
	at3	3.739 (0.912)		0.928							
	at4	3.187 (0.862)		0.810							
**Intention to use (UI)**							0.979		0.959		.958
	ui1	3.805 (1.002)		0.979							
	ui2	3.798 (0.978)		0.980							

Next, discriminant validity was verified using the Fornell–Larcker [43], cross-loading, and heterotrait-monotrait (HTMT) criteria [46]. For the Fornell–Larcker criterion, the square root of the AVE for a construct must be higher than the cross-construct correlation values. During the validation of the criterion, 1 item for perceived usefulness was excluded. Table 4 presents the correlation matrix and square root of the AVE, which shows that the Fornell–Larcker criterion was fulfilled. The cross-loading criterion was also satisfied, in which the loading value of the items on the corresponding constructs exceeded those on the other constructs. Lastly, we tested the HTMT criterion for our reflective constructs. According to Henseler et al [46], when testing the null hypothesis (H0: HTMT≥1) against the opposite hypothesis (H1: HTMT<1), if a CI contains the value 1, it indicates a lack of discriminant validity. The HTMT results for this study show that the HTMT CI does not include 1; thus, discriminant validity was established (Multimedia Appendix 1).

**Table 4:** Correlation matrix and square root of the average variance extracted. Values in italics are the square root of the AVE for the corresponding constructs.

Constructs	AC^a^	EC^b^	PE^c^	PU^d^	PD^e^	CA^f^	AT^g^	UI^h^
AC	*0.825*							
EC	0.785	*0.834*						
PE	0.660	0.713	*0.820*					
PU	0.719	0.825	0.698	*0.882*				
PD	–0.525	–0.440	–0.423	–0.398	*0.876*			
CA	0.122	0.150	0.069	0.144	0.003	*0.827*		
AT	0.711	0.731	0.663	0.727	–0.464	0.134	*0.882*	
UI	0.724	0.684	0.636	0.654	–0.518	0.083	0.802	*0.980*

^a^AC: increased accessibility.

^b^EC: enhanced care.

^c^PE: perceived ease of use.

^d^PU: perceived usefulness.

^e^PD: privacy and discomfort.

^f^CA: anxiety about COVID-19.

^g^AT: attitude toward telehealth.

^h^UI: intention to use telehealth.

### Hypothesis Testing

The structural model was developed to identify the relationships among the constructs. First, we assessed the model fit using the standardized root mean square residual (SRMR) [46] and root mean square (RMS_theta_). The SRMR value for this study was 0.061, which is less than the cut-off value of 0.08, and showed an acceptable model fit [47]. The RMS_theta_ value was 0.145, which was slightly above the recommended threshold [48], but its exact acceptable threshold values have not been determined [49].

**Figure 2:**
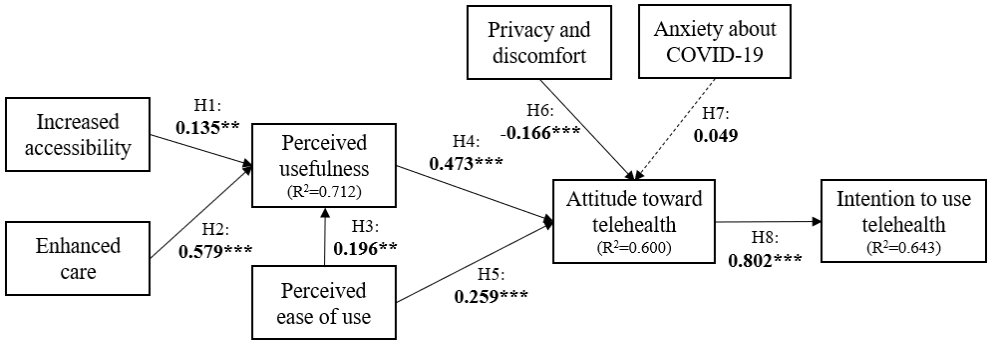
Partial least squares results and *R^2^* values (N=471). ***P<.001; **P<.01; *P<.05.

To test our hypotheses, we executed the PLS with a 500-times sampling bootstrap and evaluated the relationship between variables using path coefficient (*β*) and *t* statistics. The PLS results for the hypotheses are shown in Figure 2 and Table 5. The results show that all hypotheses, except H7, were supported. Increased accessibility and enhanced care were revealed to have a positive impact on the perceived usefulness of telehealth (H1: *t=*3.074, *P*<.01; H2: *t*=12.479, *P*<.001). Moreover, the perceived ease of use of telehealth had a positive impact on the perceived usefulness of it (*t*=5.049, *P*<.001); thus, H3 was supported. Both the perceived usefulness and the perceived ease of use of telehealth demonstrated the positive influence of attitude toward telehealth, so H4 (*t*=11.555, *P*<.001) and H5 (*t*=5.748, *P*<.001) were also supported. Privacy and discomfort about telehealth had a significantly negative influence on attitude toward telehealth (H6: *t*=4.746, *P*<.001). Meanwhile, anxiety toward COVID-19 had no significant effect on attitude toward telehealth (*t*=1.591, *P*>.05), and thus H7 was rejected. Lastly, attitude toward telehealth had a significantly positive influence on the intention to use telehealth (*t*=34.846, *P*<.001), supporting H8. The *R*^2^ value of the dependent variable of the intention to use telehealth was 0.643 (adjusted *R*^2^=0.642). This implies that 64.3% of the intention to use telehealth was elucidated by 4 precedent variables: perceived usefulness, perceived ease of use, privacy and discomfort, and anxiety.

**Table 5:** Hypothesis analysis results.

Hypothesis	Path	*β*	*t* value	P value	Comments
H1	AC^a^ → PU^b^	0.135	3.074	.002	Supported
H2	EC^c^ → PU	0.579	12.479	<.001	Supported
H3	PE^d^ → PU	0.196	5.049	<.001	Supported
H4	PU → AT^e^	0.473	11.555	<.001	Supported
H5	PE → AT	0.259	5.748	<.001	Supported
H6	PD^f^ → AT	–0.166	4.746	<.001	Supported
H7	CA^g^ → AT	0.049	1.591	.11	Not supported
H8	AT → UI^i^	0.802	34.846	<.001	Supported

^a^AC: increased accessibility.

^b^PU: perceived usefulness.

^c^EC: enhanced care.

^d^PE: perceived ease of use.

^e^AT: attitude toward telehealth.

^f^PD: privacy and discomfort.

^g^CA: anxiety about COVID-19.

^h^UI: intention to use telehealth.

Additionally, we classified participants into 2 clusters—(1) participants with chronic disease and (2) participants without chronic disease—and executed PLS analysis for each cluster. The results, shown in Multimedia Appendix 2, revealed that there was one significant difference between the clusters: among participants with chronic disease, no significant effect of increased accessibility on perceived usefulness was observed (*t*=0.142, *P*>.10). In both clusters, anxiety about COVID-19 was not significantly associated with attitude toward telehealth.

## Discussion

### Principal Findings

In this nationwide survey targeting the Korean general population, we identified factors affecting the acceptance of telehealth. Using the extended TAM, we confirmed that not only perceived usefulness and ease of use, but also increased accessibility and enhanced care, which are the characteristics of telehealth, have a positive effect on attitude toward telehealth. Privacy and discomfort were a hindrance to telehealth, and this issue calls for improvement. Unexpectedly, anxiety about COVID-19 had no significant effect on attitude toward telehealth. The neutral association between anxiety about COVID-19 and telehealth acceptance was consistent in populations with and without chronic diseases.

This study confirmed that findings from previous studies can be applied to South Korea in the pandemic context. TAM can be successfully applied to studying telehealth acceptance in the overall population. Many studies have investigated telehealth acceptance based on TAM in multiple countries such as Taiwan [50] and China [19,51], and perceived usefulness and ease of use were validated as positive factors for telehealth acceptance. The enhanced accessibility of telehealth geographically, economically, and socially are benefits of telehealth [17,52]. Along with increased accessibility, enhanced care also has significant effects on the usefulness of telehealth, which is consistent with the results of previous studies [53,54]. In terms of privacy concerns, this study confirmed the findings of previous research, which showed that such concerns negatively correlated with the intention to adopt telehealth [51].

To our knowledge, this is the first study to analyze empirically the effects of COVID-19 on telehealth acceptance. Undoubtedly, the unprecedented nature of the pandemic has induced substantial enthusiasm for telehealth worldwide [55]. Of note, no significant relationship was found between anxiety about COVID-19 and telehealth acceptance. This insignificant impact may be attributed to when the survey was conducted (July 3, 2020). At that time, the number of COVID-19 cases had decreased and remained at less than 100 from April 2 to July 25, 2020 [8]. This decline in cases may have alleviated feelings of COVID-19–related anxiety.

Moreover, our findings may indicate the possibility of telehealth use even after the pandemic. A survey targeting physician engagement with patients and telehealth experiences showed that one-fifth of clinicians expected to use telehealth more after the COVID-19 pandemic is terminated compared to before the pandemic [56]. In South Korea, about 262,000 telehealth appointments were conducted from February 24 to May 10 (3403 appointments and 142 COVID-19 cases per day on average) and about 511,000 telehealth appointments were conducted from May 10 to September 20 (3871 appointments and 97 COVID-19 cases per day on average) [57-59]. Although telehealth was allowed temporarily due to COVID-19 in South Korea, it appears that interest in and the need for telehealth have already increased. This study offers indispensable information for policymakers and health care providers on implementing appropriate telehealth services.

Since patients with chronic diseases are more susceptible to fatal outcomes due to COVID-19 than those without chronic diseases [60], we assumed that patients with chronic diseases would prefer telehealth due to anxiety about COVID-19. Our finding suggests that patients with chronic diseases may continue to use telehealth after the pandemic era due to other reasons, including enhanced care and perceived ease of use. Interestingly, the relationship between increased accessibility and perceived usefulness was also not evident in this population. It contradicted with previous findings, which demonstrated that one of the key elements of telehealth for patients with chronic diseases is increased accessibility [20,61]. Our results may be driven by the universal availability of health care use in South Korea. Kim et al [62] surveyed unmet health care needs such as economic hardship, scheduling conflict, and long waiting times among the Korean elderly, and 17.4% (economic accessibility, 9.2%; service acceptability, 6.5%; and scheduling conflict, 1.7%) of respondents answered that unmet needs exist, which is a lower percentage than those in other developed countries, including Greece (26.3%) and Canada (scheduling conflict, 54.9%; service acceptability, 42.8%; and economic accessibility, 12.7%). It may imply that telehealth is required not only for filling the gaps in the current medical supply system but also for further development in patient care.

This study also provides some guidance for telehealth service providers. First, telehealth providers should elaborate the service model to promote accessibility and health care quality. A better health care service could involve preemptive treatment before the deterioration of health [63], and consultation with general physicians after normal clinic hours [64] could be considered. Second, technology developers should couple basic technologies with a convenient user interface. Telehealth-related technologies such as data integration with electronic medical records, data connection from multiple sources [65], and biophysiological data measuring/monitoring tools should be improved [66]. Moreover, an approachable user interface should be developed to encourage patients with digital literacy to accept telehealth [67]. Third, privacy concerns and feelings of discomfort are obstacles to be overcome in telehealth. Telehealth providers should establish a privacy and security protocol corresponding to HIPAA (Health Insurance Portability and Accountability Act) or HITEC (Health Information Technology for Economic and Clinical Health) for storing, transmitting, and utilizing data to provide a private and secure telehealth service [68].

### Limitations

This study has several limitations. First, while factors associated with telehealth acceptance were included in this study, the actual behavior of adopting telehealth was not analyzed. The indirect construct of intention to use telehealth was used as a surrogate variable. Second, although the number of telehealth insurance claims were higher for those aged >30 years [69], exclusion of those in the 20-29 years age group is also a limitation of our study; this meant that users who are potentially more technologically skillful and have a greater tendency toward telehealth were omitted. Third, this study was based on cross-sectional data collected from individual surveys. Longitudinal field studies in the context of actual telehealth should be performed in the future. Fourth, we used COVID-19 anxiety measurements from a previous study that were not rigorously validated; in addition, the measures simply investigated people’s cognition and emotions related to COVID-19. It is not easy to reference well-validated measurements for anxiety in the context of a new pandemic, but it is significant that this study provides an early examination of the impact of COVID-19 on telehealth acceptance. Fifth, this study did not consider other factors that may influence telehealth acceptance. Individual, organizational, social, and legal factors such as policy, social norms, and trust in telehealth should be considered for the successful implementation of the telehealth system [70]. Lastly, because this study only included the South Korean population, it may not be generalized to other countries, which have different medical systems.

### Conclusions

Based on our extended version of TAM, this study revealed the key factors influencing user intentions and attitudes toward telehealth services in the Korean general population. Our results indicate that accessibility, enhanced care, usefulness, ease of use, and privacy and discomfort are variables affecting user intentions and attitudes in this population, while anxiety about COVID-19 did not have significant impact. This study may aid technology developers and health care decision makers to better understand the behavioral characteristics of the Korean population and lead to the tailored promotion of telehealth services after the pandemic subsides.

## Appendix

Multimedia Appendix 1 Heterotrait-monotrait (HTMT) credential interval.

Multimedia Appendix 2 Path analysis results for patients with and without chronic disease.

## Abbreviations

AVE: average variance extracted
HIPAA: Health Insurance Portability and Accountability Act
HITEC: Health Information Technology for Economic and Clinical Health
HTMT: heterotrait-monotrait
PLS: partial least squares
RMS: root mean square
SRMR: standardized root mean square residual
TAM: technology acceptance model
