# Supplementary material for: Using an Extended Technology Acceptance Model to Understand the Factors Influencing Telehealth Utilization After Flattening the COVID-19 Curve in South Korea: Cross-sectional Survey Study
Source: JMIR Med Inform. 2021 Jan 8;9(1):e25435. doi: 10.2196/25435 (PMC7801132; doi:10.2196/25435)
Supplement: Multimedia Appendix 1 [file medinform_v9i1e25435_app1.docx]

**Multimedia Appendix 1.** Heterotrait-Monotrait (HTMT) credential interval.

| Constructs | AC^a^ | EC | PE | PU | PD | CA | AT |
| --- | --- | --- | --- | --- | --- | --- | --- |
| EC^b^ | 0.865~  0.936 |  |  |  |  |  |  |
| PE^c^ | 0.698~  0.831 | 0.755~  0.865 |  |  |  |  |  |
| PU^d^ | 0.783~  0.884 | 0.902~  0.971 | 0.750~  0.859 |  |  |  |  |
| PD^e^ | 0.521~  0.726 | 0.398~  0.598 | 0.389~  0.600 | 0.345~  0.562 |  |  |  |
| CA^f^ | 0.071~  0.258 | 0.079~  0.294 | 0.069~  0.194 | 0.078~  0.288 | 0.040~  0.134 |  |  |
| AT^g^ | 0.749~  0.869 | 0.735~  0.867 | 0.657~  0.817 | 0.734~  0.876 | 0.411~  0.622 | 0.063~  0.266 |  |
| UI^h^ | 0.745~  0.862 | 0.663~  0.805 | 0.617~  0.762 | 0.629~  0.781 | 0.476~  0.670 | 0.028~  0.212 | 0.816~  0.893 |

^a^ Increased accessibility

^b^ Enhanced care

^c^ Perceived ease of use

^d^ Perceived usefulness

^e^ Privacy and discomfort

^f^ Covid-19 anxiety

^g^ Attitude toward telehealth

^h^ Use intention to telehealth
