# Supplementary material for: Using an Extended Technology Acceptance Model to Understand the Factors Influencing Telehealth Utilization After Flattening the COVID-19 Curve in South Korea: Cross-sectional Survey Study
Source: JMIR Med Inform. 2021 Jan 8;9(1):e25435. doi: 10.2196/25435 (PMC7801132; doi:10.2196/25435)
Supplement: Multimedia Appendix 2 [file medinform_v9i1e25435_app2.docx]

**Multimedia Appendix 2.** The result of path analysis for chronic and non-chronic disease patient.

| Path | Target | $\beta$ | t-value | p-value | Comments |
| --- | --- | --- | --- | --- | --- |
| AC^a^ → Pu^b^ | Chronic | 0.011 | 0.142 | > .1 | Not significant |
|  | Non-chronic | 0.196 | 3.780 | < .001 | Significant |
| EC^c^ → PU | Chronic | 0.655 | 7.997 | < .001 | Significant |
|  | Non-chronic | 0.547 | 9.620 | < .001 | Significant |
| PE^d^ → PU | Chronic | 0.232 | 3.721 | < .001 | Significant |
|  | Non-chronic | 0.177 | 3.581 | < .001 | Significant |
| PU → AT^e^ | Chronic | 0.438 | 6.217 | < .001 | Significant |
|  | Non-chronic | 0.491 | 8.991 | < .001 | Significant |
| PE → AT | Chronic | 0.284 | 3.869 | < .001 | Significant |
|  | Non-chronic | 0.248 | 4.162 | < .001 | Significant |
| PD^f^ → AT | Chronic | -0.199 | 3.455 | < .001 | Significant |
|  | Non-chronic | -0.153 | 3.640 | < .001 | Significant |
| CA^g^ → AT | Chronic | 0.074 | 1.325 | > .1 | Not significant |
|  | Non-chronic | 0.032 | 0.764 | > .1 | Not significant |
| AT → UI^h^ | Chronic | 0.739 | 17.162 | < .001 | Significant |
|  | Non-chronic | 0.825 | 34.270 | < .001 | Significant |

^a^ Increased accessibility

^b^ Perceived usefulness

^c^ Enhanced care

^d^ Perceived ease of use

^e^ Attitude toward telehealth

^f^ Privacy and discomfort

^g^ Covid-19 anxiety

^h^ Use intention to telehealth
